# Spatial and temporal distribution of *Culex* and *Aedes* mosquitoes in Ghana

**DOI:** 10.46471/gigabyte.170

**Published:** 2025-12-15

**Authors:** Yaw Akuamoah-Boateng, Christopher Mfum Owusu-Asenso, Anisa Abdulai, Abdul Rahim Mohammed Sabtiu, Isaac Kwame Sraku, Sebastian Kwo Egyin Mensah, Faustina Adobea Owusu, Abena Ahema Ebuako, Godfred Amoateng, Bright Churchill Obeng, Richard Tettey Doe, Emmanuel Nana Boadu, Akua Aboagyewaa Appiah, Grace Arhin Danquah, Nutifafa Efui Abusah, Dhikrullahi Bunkunmi Shittu, Gabriel Akosah-Brempong, Cosmos Manwovor-Anbon Pambit Zong, Daniel Kodjo Halou, Osei Kwaku Akuoko, Cornelia Appiah-Kwarteng, Yaw Asare Afrane

**Affiliations:** ^1^ Centre for Vector-Borne Disease Research, Department of Medical Microbiology, University of Ghana Medical School, Korle-Bu, Accra, Ghana; ^2^ African Regional Postgraduate Programme in Insect Sciences (ARPPIS), Department of Animal Biology and Conservation Science, College of Basic and Applied Sciences, University of Ghana, Accra, Ghana; ^3^ Department of Vector Biology, Liverpool School of Tropical Medicine, UK; ^4^ Department of Parasitology, Noguchi Memorial Institute for Medical Research, College of Health Sciences, University of Ghana, Legon, Accra, Ghana; ^5^ School of Veterinary Medicine, University of Ghana, Legon, Accra, Ghana

## Abstract

In Africa, *Culex* is an important vector that transmits West Nile virus, whilst *Aedes* mosquitoes transmit dengue, yellow fever, chikungunya, and Zika. However, very limited data is available on their bionomics and ecology. Here, we provide data on the abundance and distribution of *Culex* and *Aedes* mosquitoes in Ghana between 2017 and 2025. We collected 39,761 *Culex* and 6,047 *Aedes* mosquitoes using various mosquito-trapping tools. Both vectors were predominantly observed outdoors. *Aedes aegypti* was the most dominant *Aedes* vector observed in Ghana. The invasive *Aedes albopictus* was sampled in 2023, whereas *Aedes vittatus* was observed in Accra. Our data provides important information to support vector surveillance, ecological risk assessments, and integrated vector-management strategies.

## Data description

### Background and context

*Culex* mosquitoes are important vectors of emerging arboviruses such as West Nile virus and Japanese encephalitis [1, 2]. Also, *Aedes* mosquitoes are the primary vectors of dengue, Zika, chikungunya, and yellow fever viruses, posing a significant public health threat globally [3]. Invasion of *Aedes albopictus* mosquitoes in the West African sub-region has coincided with multiple outbreaks of dengue fever [4, 5]. However, the distribution of *Culex* and *Aedes* mosquitoes and their role in transmitting these pathogens remains understudied. This gap limits our ability to map the distribution of *Culex* and *Aedes* species across varying ecological zones to plan targeted vector-control interventions and management strategies. To help bridge this gap, it is important to study the ecology and bionomics of *Culex* and *Aedes* mosquitoes at fine and operational spatiotemporal scales.

In this paper, we present a dataset on the spatial and temporal distribution and ecology of *Culex* and *Aedes* mosquitoes. This resource may guide the targeted control and elimination of arboviral vectors in Ghana. Also, it will help identify potential hotspots for *Culex-* and *Aedes*-borne diseases in relation to urbanization, environmental change, and human exposure.

## Methods

### Study sites

**Figure 1.**
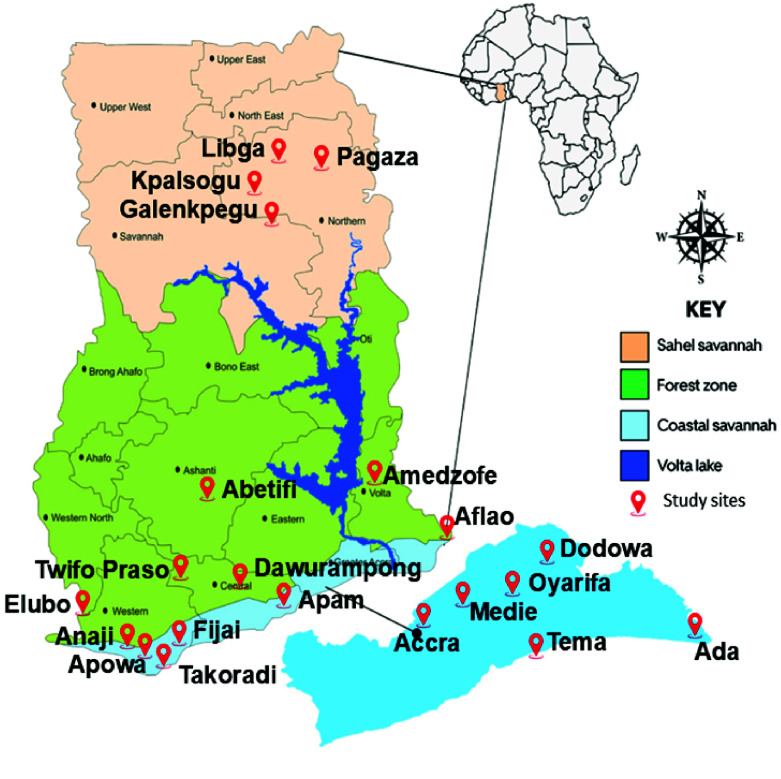
Map of Ghana showing the study sites.

Mosquito sampling was conducted in twenty-two communities in different ecological settings in Ghana, comprising the Coastal Savannah (Accra, Ada, Aflao, Apam, Dawurampong, Dodowa, Elubo, Medie, Oyarifa, Takoradi, Tema, Anaji, Apowa, Fijai), Forest (Konongo, Twifo Praso, Abetifi, Amedzofe), and Sahel Savannah (Kpalsogu, Pagaza, Libga, Galenkpegu) (Figure 1). The Coastal Savannah is in the south of Ghana and it experiences a bimodal rainfall pattern. The Coastal savannah rainy seasons occur between April and June, and between September and October. The dry season in the Coastal Savannah typically runs from November to March. The Forest zone in the middle of Ghana also experiences a bimodal rainfall pattern with rainy periods between March and June, and between September and October, with a dry season between November and February. Finally, the Sahel Savannah in the north of Ghana has a unimodal rainfall pattern from May to November, whereas the dry season (December–April) sees temperatures rise as high as 42 °C.

### Mosquito sampling methods

Mosquito collections were performed at two time points annually (Dry and Rainy seasons) from 2017 to 2025. Resting adult *Culex* and *Aedes* mosquitoes were collected using Prokopack aspirations, Pyrethrum Spray Catches, Clay pots, and Pit traps [6]. Host-seeking adult *Culex* and *Aedes* mosquitoes were collected using Human Landing Catches (HLC) [7], Biogent Sentinel traps (BG) and Light traps (CDC miniature light trap). At each sampling site, sixteen houses were randomly selected for HLC, BG traps, and light trap collections. Twenty houses were randomly selected for Prokopack aspirations. Samples were collected both indoors and outdoors. Clay pot traps and Pit trap collections were carried out as described by Odiere *et al.* [6] and Mawejje *et al.* [8], respectively. Four houses were randomly selected for clay pot collection, and two pit traps per community.

### Data validation and quality control

Collected mosquito samples were morphologically sorted and identified as *Culex* or *Aedes* mosquitoes. *Aedes* mosquitoes were morphologically identified using well-established morphological keys [9]. *Culex* mosquitoes were not identified to species. Data was thoroughly screened and automatically validated using the Integrated Publishing Toolkit of Global Biodiversity Information Facility (GBIF) [10].

## Results

### Abundance and distribution of *Culex* mosquitoes in Ghana

A total of 39,761 *Culex* mosquitoes were collected across the study sites in different ecological zones of Ghana (Table 1). *Culex* mosquito abundance was higher in the rural communities (55.47% of all our collected Culex mosquitoes; 22,056/39,761) than in the urban (34.71%; 13,801/39,761) and peri-urban (9.42%; 3,904/39,761) communities. A high proportion of the *Culex* mosquitoes collected were caught outdoors (80.72%; 32,094/39,761), whereas 19.28% (7,667/39,761) were caught indoors. A Chi-square test of independence showed a statistically significant difference in vector collection between indoor and outdoor sites across the urban, rural, and peri-urban sites (*X*^2^ = 5800, *df* = 2, *p* < 0.001).

**Table 1:** *Culex* mosquito distribution across Ghana.

Sampling site	Site classification	Ecological zone	Location		Total (*n*)
			Indoor (*n*)	Outdoor (*n*)	
Abetifi	Peri-urban	Forest	0	59	59
Accra	Urban	Coastal	0	12,939	12,939
Ada	Rural	Coastal	5,148	8,579	13,727
Aflao	Urban	Coastal	429	192	621
Amedzofe	Rural	Forest	0	2	2
Apam	Rural	Coastal	41	36	77
Dawurampong	Rural	Coastal	3	1	4
Dodowa	Rural	Coastal	732	1,562	2,294
Elubo	Urban	Coastal	15	55	70
Galenkpegu	Rural	Sahel-savannah	10	2	12
Konongo	Rural	Forest	188	650	838
Kpalsogu	Rural	Sahel-savannah	475	1,333	1,808
Libga	Rural	Sahel-savannah	256	1,079	1,335
Medie	Peri-urban	Coastal	0	2,478	2,478
Oyarifa	Peri-urban	Coastal	0	1,297	1,297
Pagaza	Rural	Sahel-savannah	320	1,443	1,763
Takoradi	Urban	Coastal	0	235	235
Tema	Urban	Coastal	0	6	6
Twifo Praso	Rural	Forest	50	120	170
**Total**			**7,667**	**32,094**	**39,761**

**Figure 2.**
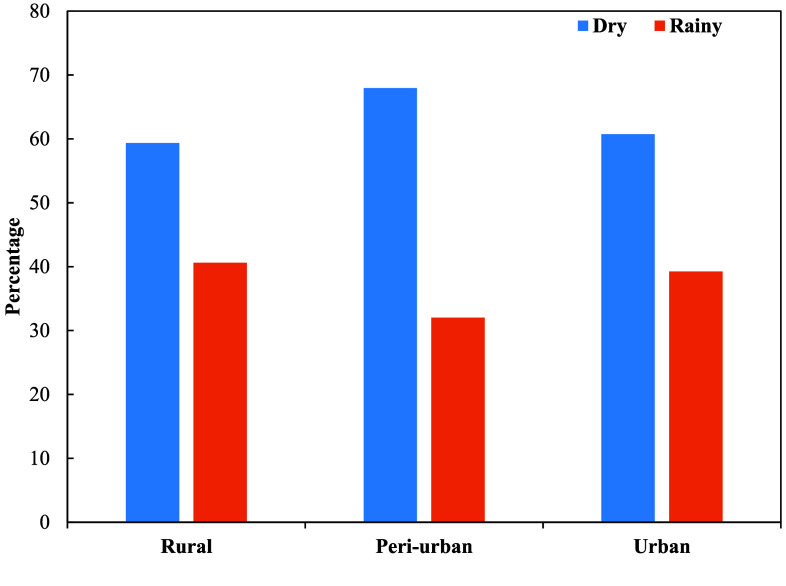
Seasonal distribution of *Culex* mosquitoes in Ghana.

*Culex* mosquito abundance was higher during the dry season (60.69%; 24,131/39,761) compared to the rainy season (39.31%; 15,630/39,761) (Figure 2). A statistically significant difference in the seasonal distribution of *Culex* mosquitoes was observed across the urban, rural, and peri-urban sites (Chi-square test, *X*^2^(2) = 103.20, *p* < 0.001).

### Abundance and distribution of *Aedes* mosquitoes in Ghana

**Figure 3.**
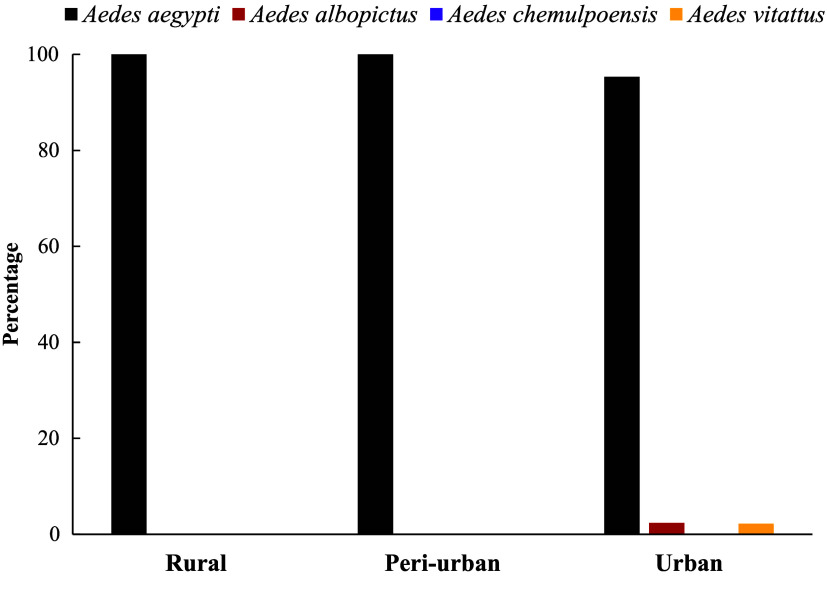
*Aedes* mosquito species distribution across Ghana.

A total of 6,047 *Aedes* mosquitoes were collected across sites in different ecological zones of Ghana (Table 2). The *Aedes* mosquitoes were sampled predominantly outdoors (88.03%; 5,323/6,047), whereas 11.97% (724/6,047) were sampled indoors. *Aedes aegypti* mosquitoes were the most abundant *Aedes* sampled in all communities (5,817/6,047). *Aedes vittatus* mosquitoes (108/6,047) were found in Accra. *Aedes albopictus* (116/6,047) and *Aedes chemulpoensis* (6/6,047) mosquitoes were found in Takoradi, an urban settlement (Figure 3). The invasive *Aedes albopictus* was first sampled in 2023 [11]. A significant association was observed between the urban-rural continuum and mosquito collection location (*X*^2^(1) = 989.60, *p* < 0.001). *Aedes* mosquitoes in the rural areas were more likely to be collected indoors (39.6%), whereas those in the urban areas were mostly collected outdoors (94.3%).

**Table 2:** *Aedes* mosquito abundance and distribution across Ghana.

Site	Site classification	Ecological zone	Location		Total (*n*)
			Indoor (*n*)	Outdoor (*n*)	
Abetifi	Peri-urban	Forest	2	0	2
Accra	Urban	Coastal	129	2,695	2,824
Ada	Rural	Coastal	270	251	521
Aflao	Urban	Coastal	7	19	26
Anaji	Urban	Coastal	0	114	114
Apam	Rural	Coastal	31	27	58
Apowa	Urban	Coastal	47	396	443
Dawurampong	Rural	Coastal	12	2	14
Dodowa	Rural	Coastal	17	56	73
Elubo	Urban	Coastal	1	5	6
Fijai	Urban	Coastal	0	1	1
Galenkpegu	Rural	Sahel-savannah	0	1	1
Konongo	Rural	Forest	11	13	24
Kpalsogu	Rural	Sahel-savannah	9	63	72
Libga	Rural	Sahel-savannah	66	146	212
Pagaza	Rural	Sahel-savannah	17	108	125
Takoradi	Urban	Coastal	70	1,231	1,301
Tema	Urban	Coastal	29	194	223
Twifo Praso	Rural	Forest	6	1	7
**Total**			**724**	**5,323**	**6,047**

**Figure 4.**
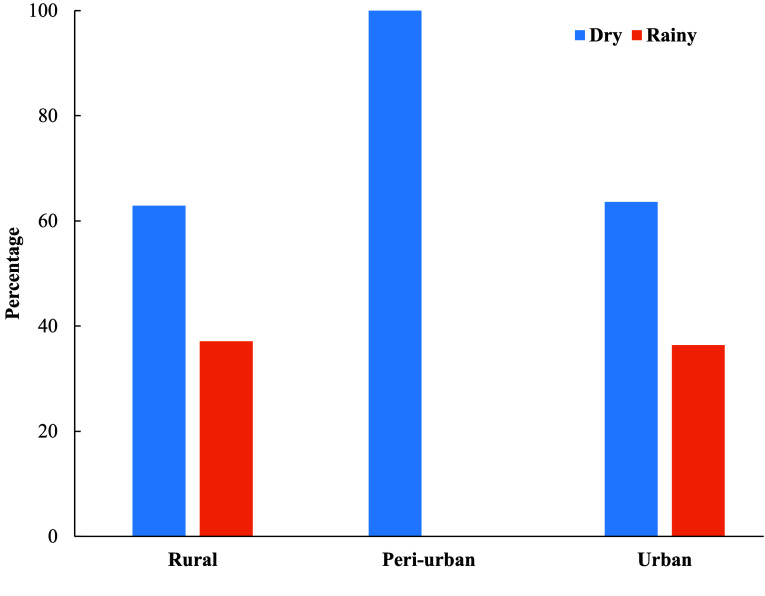
Seasonal distribution of *Aedes* mosquitoes in Ghana.

More *Aedes* mosquitoes were collected during the dry season than during the rainy season in all ecological zones. In the rural sites, 62.91% (697/1,108) of *Aedes* mosquitoes were sampled in the dry season, whereas 37.09% (411/1,108) were sampled in the rainy season. In the peri-urban sites, 100% (2/2) were sampled in the dry season. Similarly, in the urban sites, 63.62% (3,141/4,937) of the *Aedes* mosquitoes caught were sampled during the dry season, whereas 36.38% (1,706/4,937) were caught during the rainy season (Figure 4).

## Re-use potential

The two datasets presented here offer valuable insights into the distribution of *Culex* and *Aedes* mosquito species in Ghana. Researchers and public health professionals can use this dataset to enhance their understanding of the ecology and distribution of *Culex* and *Aedes* mosquitoes. The information in the dataset can serve as a resource for studies assessing transmission risks, vector control strategies, disease surveillance, and a broader understanding of the ecology of *Culex* and *Aedes* mosquitoes across Ghana’s various ecological zones.

## Data Availability

The two datasets described here are available via the GBIF repository [12, 13].

## Editor’s note

This paper is part of a series of Data Release articles working with GBIF and supported by TDR, the Special Program for Research and Training in Tropical Diseases, hosted at the World Health Organization [14].

## Abbreviations

BG, Biogent Sentinel traps; CDC, Centers for Disease Control and Prevention; GBIF: Global Biodiversity Information Facility; HLC, Human Landing Catches.
